# Interfacial exchange coupling and magnetization reversal in perpendicular [Co/Ni]_**N**_**/**TbCo composite structures

**DOI:** 10.1038/srep10863

**Published:** 2015-06-15

**Authors:** M. H. Tang, Zongzhi Zhang, S. Y. Tian, J. Wang, B. Ma, Q. Y. Jin

**Affiliations:** 1 Shanghai Engineering Research Center of Ultra-precision Optical Manufacturing, and Key Laboratory of Micro and Nano Photonic Structures (Ministry of Education), Department of Optical Science and Engineering, Fudan University, Shanghai, 200433, China; 2 Department of Physics, Ningbo University, Ningbo 315211, China

## Abstract

Interfacial exchange coupling and magnetization reversal characteristics in the perpendicular heterostructures consisting of an amorphous ferrimagnetic (FI) Tb_*x*_Co_100–*x*_ alloy layer exchange-coupled with a ferromagnetic (FM) [Co/Ni]_N_ multilayer have been investigated. As compared with pure Tb_*x*_Co_100–*x*_ alloy, the magnetization compensation composition of the heterostructures shift to a higher Tb content, implying Co/Ni also serves to compensate the Tb moment in TbCo layer. The net magnetization switching field *H*_c⊥_ and interlayer interfacial coupling field *H*_ex_, are not only sensitive to the magnetization and thickness of the switched Tb_*x*_Co_100–*x*_ or [Co/Ni]_N_ layer, but also to the perpendicular magnetic anisotropy strength of the pinning layer. By tuning the layer structure we achieve simultaneously both large *H*_c⊥_ = 1.31 T and *H*_ex_ = 2.19 T. These results, in addition to the fundamental interest, are important to understanding of the interfacial coupling interaction in the FM/FI heterostructures, which could offer the guiding of potential applications in heat-assisted magnetic recording or all-optical switching recording technique.

In the past years, extensive work has been devoted to the heterostructures with a ferromagnetic (FM) /antiferromagnetic (AFM) exchange coupling interface^1^,^2^,^3^,^4^,^5^,^6^. The exchange bias effect^7^, arising from the interfacial unidirectional anisotropy and displaying a shift along the magnetic field axis of the magnetic hysteresis loop, has been employed in giant magnetoresistance (GMR) devices for magnetic data storage applications^8^,^9^,^10^. Recently, with great demand for increasing storage density, new writing technique such as heat-assisted magnetic recording (HAMR)^10^,^11^,^12^ and all-optical switching (AOS)^13^,^14^ have received much attention, which intrigues great interest in seeking perpendicular exchange-coupled heterostructures with strong and temperature (*T*) sensitive interfacial AFM exchange coupling. However, in previous studies, most of the exchange-coupled heterostructures with perpendicular magnetic anisotropy (PMA) are restricted to a FM layer antiferromagnetically coupled with an AFM layer such as MnIr or FeMn, for which the room temperature (RT) coupling field (*H*_ex_) is usually below 0.1 T^3^,^4^,^5^,^6^, apparently cannot meet the practical requirements of future high density data storage.

It is known that in ferrimagnetic (FI) rare earth-transition metal (RE-TM) alloy films, there are two types of pair interactions: antiparallel exchange between the RE-TM moments and parallel exchange of the TM moments themselves, both interactions would greatly enhance the coupling strength^12^,^14^,^15^,^16^,^17^,^18^. Moreover, amorphous ferrimagnetic RE-TM films such as TbCo or TbFe can exhibit strong PMA when the antiferromagnetically coupled magnetic moments of RE and TM are nearly balanced by controlling the element composition or measurement temperature^19^. As a result, a perpendicular heterostructure consisting of a FM layer in contact with a FI alloy layer can owns not only low stray field, but also strong, tunable, and *T*-dependent interfacial coupling and net magnetization switching fields (*H*_c⊥_)^20^,^21^. Such FI-based heterostructures might possess potential applications in HAMR and AOS, since its large and temperature-sensitive *H*_c⊥_ and *H*_*ex*_ can be used to store information while the strongly coupled FM layer can improve the properties of readout. Therefore, understanding and clarifying the related coupling and switching mechanism in the FM/FI heterostructures should be of great importance for ultrahigh density recording in these new storage techniques.

Due to the great potential applications in data storage technology, in recent years some research work has been performed regarding the FM/FI composite structures^12^,^15^,^16^,^17^. For instance, S. Romer *et al.* investigated the temperature dependence of large exchange bias effect in TbFe/[Co/Pt] system^12^. The dependence of interfacial exchange coupling on the stoichiometry of TbFe layer and repetition numbers of Co/Pt was analyzed by C. Schubert *et al.*^17^. However, the net magnetization switching properties were not well analyzed in these studies. Moreover, except Co/Pt (Pd) multilayer, no other FM layer material has been employed. In our previous work, the [Co/Ni]_N_ multilayer has been employed to couple with TbCo as the reference layer of perpendicular spin valves, by which we have achieved high GMR signal and large switching plateau^20^. The Co/Ni multilayer, which owns relatively higher spin polarization and smaller Gilbert damping factor than Co/Pt, has been considered as a potential material in MRAMs for high spin torque efficiency^22^. In order to thoroughly understand the interfacial exchange coupling, magnetization reversal, and their relationship in FM/FI heterostructures, in this work we have fabricated several series of samples of glass /Ta(3) /Cu(3) /[Co(0.28)/Ni(0.58)]_N_ /Co(*t*_Co_) /Tb_*x*_Co_100–*x*_(*t*) /Ta(5) (layer thickness in unit of nm). The influences of Tb contents *x*, Co/Ni repetition number N, and thicknesses (*t*_Co_ and *t*) of the additional Co and TbCo layers will be discussed. Note that for all these samples the easy axes of both the FI TbCo and FM [Co/Ni]_N_ layers are maintained perpendicular to the film plane.

## Results

**Figure 1:**
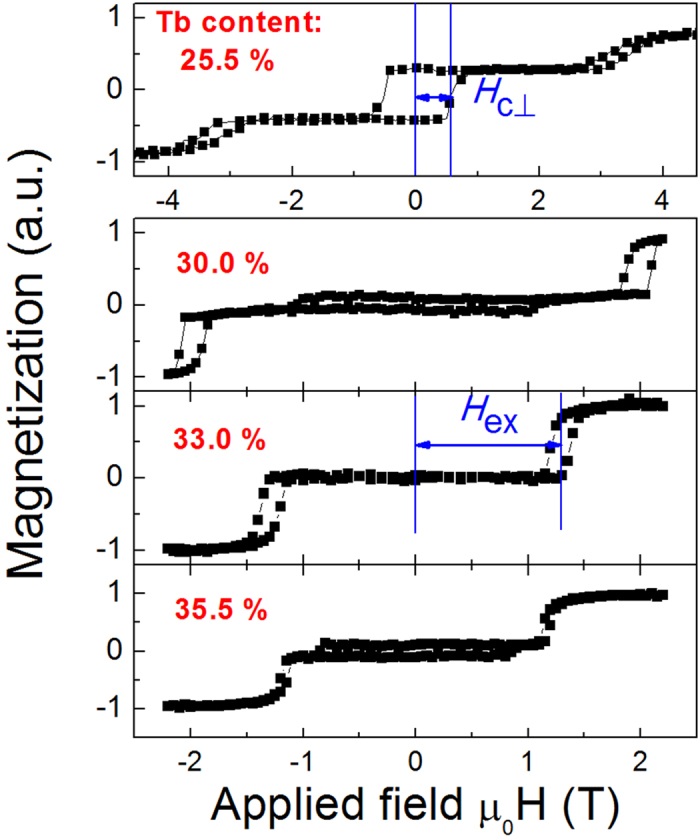
Magnetic hysteresis loops of different Tb contents. The out-of-plane magnetic hysteresis loops measured by PPMS for samples of [Co0.28/Ni0.58]_5_/ Tb_*x*_Co_100–*x*_ (12) with various Tb contents, in which the *H*_c⊥_ and *H*_ex_ are defined.

Figure 1 displays the out-of-plane magnetic hysteresis loops measured by Physical Property Measurement System (PPMS) for the heterostructure samples of [Co/Ni]_5_/Tb_*x*_Co_100–*x*_ (12) with various Tb content *x*. As defined in the loops, the magnetic coercivity *H*_c⊥_ in the central loop corresponds to the total net magnetic moment switching, while the antiferromagnetic coupling field *H*_*ex*_ denotes the magnetization switching of either TbCo or Co/Ni layer that has lower magnetic moment. Clearly, for different *x* the loops exhibit different *H*_*ex*_ and *H*_c⊥_. Especially for *x* = 33.0%, no central switching loop can be detected, indicating that the magnetization of 12-nm thick Tb_33_Co_67_ layer is balanced with that of Co/Ni and thus the sample has nearly zero net magnetic moment.

**Figure 2:**
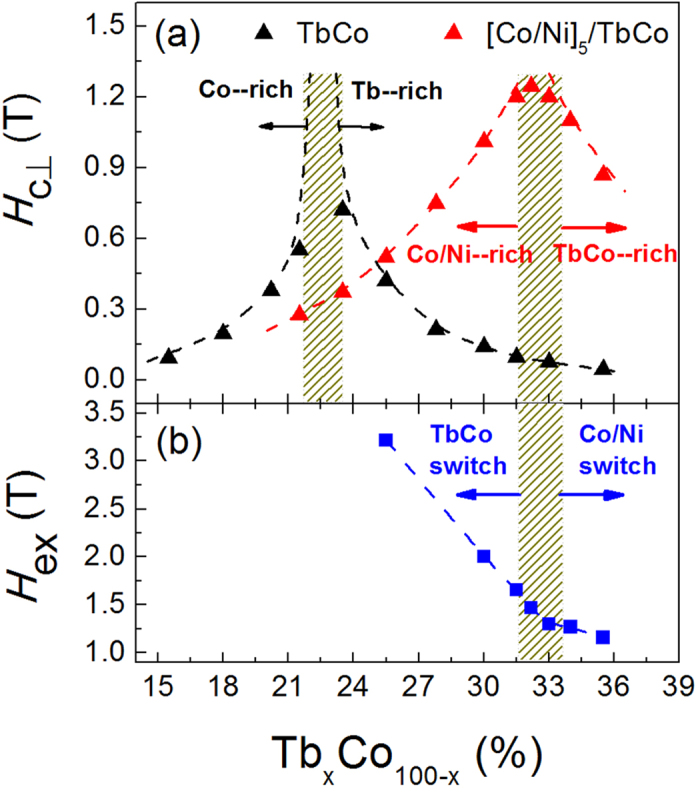
The Tb content influence on the *H*c_⊥_ and *H*_ex_. The Tb content dependences of perpendicular coercive field *H*_c⊥_ (**a**) and exchange coupling field *H*_ex_ (**b**) for the heterostructure samples of [Co0.28/Ni0.58]_5_/ Tb_*x*_Co_100–*x*_ (12). The *H*_c⊥_ values for pure TbCo alloy are also shown in (**a**).

In order to clearly see the variation trends, Tb content dependence of *H*_c⊥_ and *H*_*ex*_ are shown in Fig. 2, the *H*_c⊥_ values of pure Tb_*x*_Co_100–*x*_ are also given for comparison. Note that, for the pure TbCo alloy film, the *H*_c⊥_ firstly increases with increasing Tb content, at *x* ≈ 22% it starts to decrease. It is noticed that the largest *H*_c⊥_ occurs when the magnetic moments of Tb and Co are approaching compensated according to the inverse relation to the magnetization^23^,
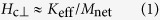
where *K*_eff_ and *M*_net_ denote the effective magnetic anisotropy and net saturation magnetization of the heterostructure, respectively. As a result, for our 12-nm thick TbCo film, the magnetization compensation composition at RT is approximately 22%. Interestingly, for the perpendicularly exchange-coupled [Co/Ni]_5_/TbCo composite film, a similar relationship between Tb content and *H*_c⊥_ happens. Nevertheless, the RT compensation composition has been moved to a higher Tb content of *x* ≈ 33%, verifying that the Co/Ni atoms also serve to compensate the magnetic moment of Tb atoms in the Tb-rich TbCo layer^20^,^21^. Therefore, for *x* < 33%, the heterostructure is [Co/Ni]_5_ rich in magnetic moment and the *H*_ex_ comes from magnetization switching of the TbCo layer. On the contrary, for *x* > 33% it becomes TbCo rich and the *H*_ex_ corresponds to the switching field of Co/Ni multilayer. As shown in Fig. 2(b), the *H*_*ex*_ reaches a value as high as 3.22 T at *x* = 25.5%, significantly larger than the exchange field observed in normal FM/AFM systems. With the increase of *x*, it firstly decreases rapidly until *x* reaches the compensation point, after that it becomes nearly stable. The observed *H*_*ex*_ tendency of the heterostructure can be well interpreted by the following formula^17^,^24^,
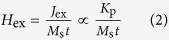
where *J*_ex_ is the interlayer coupling strength, *M*_s_ and *t* represent the saturation magnetization and thickness of the switched layer, while *K*_p_ is the magnetic anisotropy energy of the pinning layer, respectively. At *x* < 33%, the TbCo layer is switched since it has a lower moment than the Co/Ni layer, so the strong decrease of *H*_ex_ with the increase of *x* is mainly caused by the increased magnetization of TbCo layer. At *x* > 33%, the Co/Ni layer is switched, further increase of Tb content will only affect the *K*_p_ of TbCo slightly^25^, thus giving rise to the observed slight reduction of *H*_ex_.

**Figure 3:**
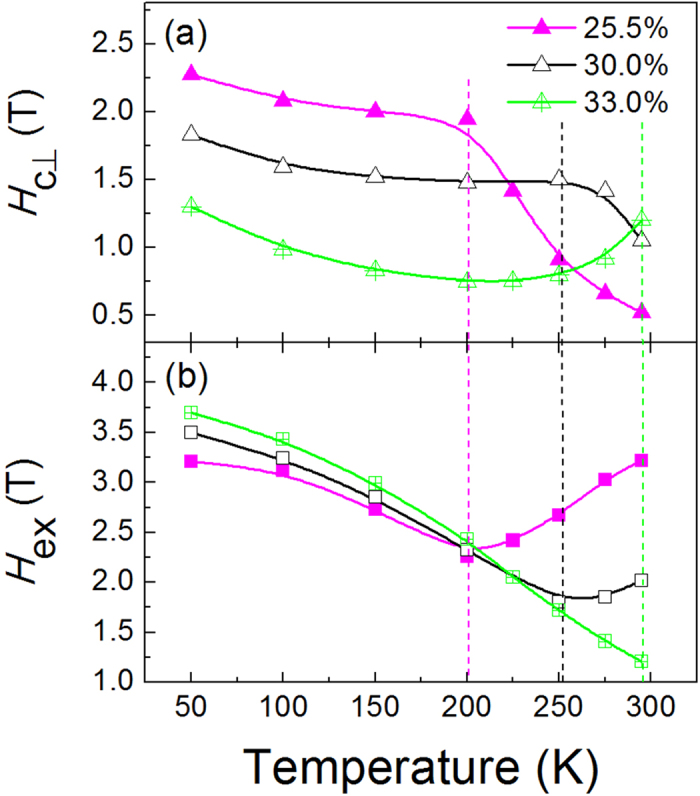
Temperature dependence of *H*c_⊥_ and *H*_ex_. (**a**) The perpendicular coercive field *H*_c⊥_ and (**b**) exchange coupling field *H*_ex_ as a function of measurement temperature for Tb contents of *x* = 25.5%, 30.0%, and 33.0%.

Considering that the Tb moment enlarges much more rapidly than the transition metal as the measurement temperature decreases, the *H*_c⊥_ and *H*_ex_ will have different temperature-dependent variation trends for samples with different Tb contents, which can be clearly seen in Fig. (3). As shown in Fig. 3(a), the *H*_c⊥_ value for *x* = 25.5% decreases slowly with the increase of temperature when *T* is below 200 K, above which it drops dramatically. Here *T* = 200 K is the transition point of magnetization compensation temperature (*T*_Mcomp_) for the sample of *x* = 25.5%, i.e. the sample is Co/Ni rich at *T* > *T*_Mcomp_ and TbCo rich as *T* < *T*_Mcomp_. According to our analyses in the preceding parts, maximum *H*_c⊥_ should take place at the *T*_Mcomp_ where magnetic moments are compensated. However, the *H*_c⊥_ value keeps increasing as *T* decreases from 200 K to 50 K, we attribute this increase to the enhanced PMA strength of TbCo pinning layer at reduced temperatures. For the *x* = 30.0% sample the temperature dependence of *H*_c⊥_ is similar to that of *x* = 25.5% case, except that it has a higher *T*_Mcomp_ of about 260 K. However, for the sample of *x* = 33.0% which has a *T*_Mcomp_ of RT, the varying trend of *H*_c⊥_ is distinctly different from the other two samples. Instead of a monotonic slow increase as *T* decreases from *T*_Mcomp_ = RT, the *H*_c⊥_ value firstly decreases and subsequently increases after reaching a minimum at about 200 K. We attribute such behavior to the combined action of enhanced PMA strength and net magnetic moment. The initial decrease of *H*_c⊥_ originates from the enlarged uncompensated moment of the heterostructure due to the much rapidly increased Tb moment in TbCo layer, whereas with further decreasing temperature the PMA enhancement plays a dominant role which leads to the slow increasing behavior, similar to the varying trend of samples of *x* = 25.5% and 30.0% at T < 200 K. Interestingly, as shown in Fig. 3(b), the *H*_ex_ value at *T* = *T*_Mcom_ is always the smallest for all the three samples. The fast increase of *H*_ex_ with *T* at *T* > *T*_Mcomp_ can be ascribed to the reduced magnetization of TbCo switched layer. Nevertheless, at *T* < *T*_Mcomp_ the switching field change of Co/Ni layer is very likely related to the enhanced PMA of TbCo pinning layer.

**Figure 4:**
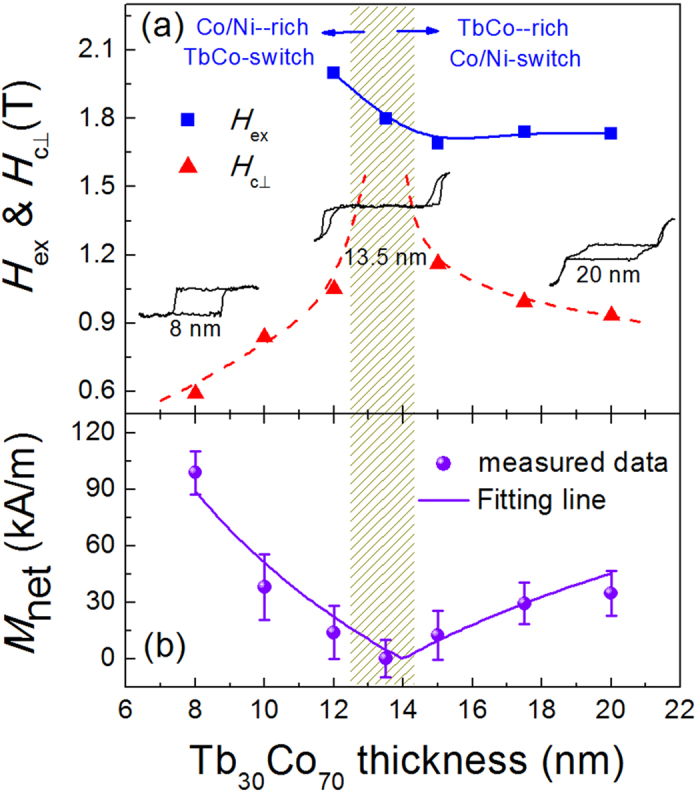
The influence of Tb_30_Co_70_ thickness on the magnetic properties. (**a**) The perpendicular coercive field *H*_c⊥_ and exchange coupling field *H*_ex_, and (**b**) remanent net magnetization *M*_net_ as a function of Tb_30_Co_70_ thickness for samples of [Co0.28/Ni0.58]_5_/ Tb_30_Co_70_ (*t*). Insets: Magnetic hysteresis loops for *t* = 8, 13.5, and 20 nm.

In addition to the Tb content, the TbCo layer thickness also plays an important role on the magnetization switching of [Co/Ni]_5_/TbCo heterostructures. Figure 4(a) shows the *H*_c⊥_ and *H*_ex_ values for samples with a fixed Tb content of *x* = 30.0% but various Tb_30_Co_70_ layer thicknesses. Three representative out-of-plane magnetic loops of *t* = 8.0, 13.5, and 20 nm, measured by Vibrating Sample Magnetometer (VSM), are inserted in Fig. 4(a). With the increase of Tb_30_Co_70_ thickness, we find the *H*_c⊥_ varies also non-monotonically. It firstly increases with increasing *t* and then begins to decrease at *t* = 13.5 nm, which means that *t* ≈ 13.5 nm is the magnetization compensation thickness for the [Co0.28/Ni0.58]_5_/Tb_30_Co_70_ (*t*) heterostructure. Meanwhile, no central switching loop takes place from the magnetic hysteresis loop of *t* = 13.5 nm sample, confirming that the total net magnetic moment is compensated. Accompanied with the change of *H*_c⊥_, the exchange coupling field *H*_ex_ also varies but in a different way. At *t* <13.5 nm, the sample is Co/Ni rich, so the coupling field comes from the TbCo layer switching, which certainly increases with the decrease of TbCo layer thickness. As soon as *t* is over 13.5 nm, the Co/Ni layer with fixed magnetization and thickness will be switched. Therefore, owing to the increased PMA of thicker TbCo layer, the *H*_ex_ exhibits a weak increase. Note that the *H*_ex_ value for the samples of *t* <12 nm is not given here because it is far beyond the highest magnetic field of 2.2 T supplied by our VSM. Figure 4 (b) shows the remanent net magnetization (*M*_net_) values as a function of Tb_30_Co_70_ thickness. As expected, starting from the compensation thickness, the *M*_net_ increases from zero towards both thicker and thinner Tb_30_Co_70_ layers. By using the following equation of *M*_net_ = (*M*_s–FM_*t*_FM_-*M*_s–FI_*t*_FI_)/(*t*_FM_ + *t*_FI_), we obtain the Co/Ni magnetization of *M*_s–FM_ = 596 ± 45 kA/m and Tb_30_Co_70_ of *M*_s–FI_ = 183 ± 15 kA/m, which are in good agreement with the measured values (604 kA/m for [Co0.28/Ni0.58]_5_ and 176 kA/m for Tb_30_Co_70_). Based on these results, we calculate the interfacial coupling strength *J*_ex_ is up to 4.4 ± 0.3 mJ/m^2^, such magnitude is comparable to the value found in other exchange-coupled FI/FM structures^12^,^17^, but greatly higher than that of the FM/AFM systems.

**Figure 5:**
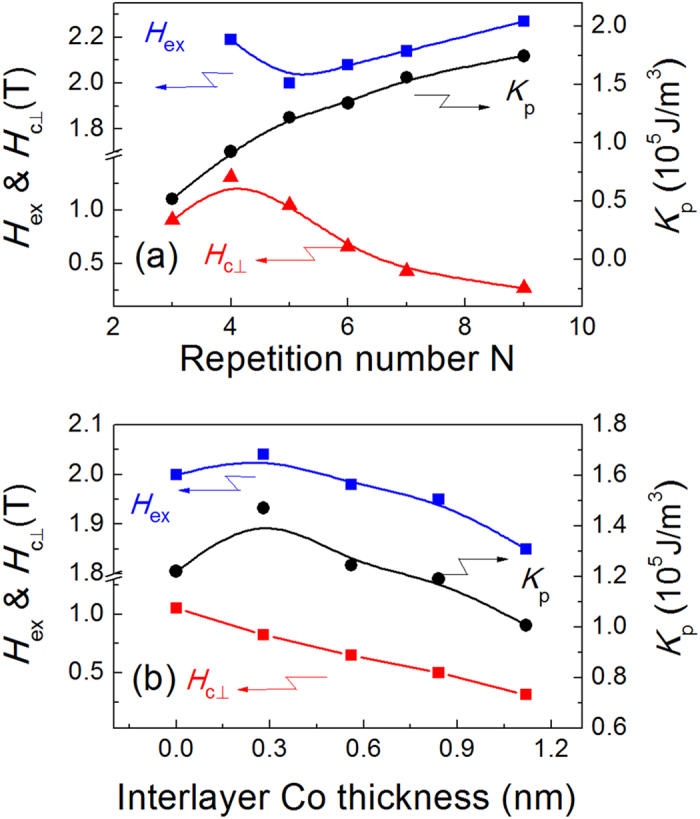
The PMA strength effect on the magnetic properties. Dependences of *H*_c⊥_, *H*_ex_, and magnetic anisotropy energy *K*_p_ of the FM layer on (**a**) the repetition number N for the samples of [Co0.28/Ni0.58]_N_/ Tb_30_Co_70_ (12) and (**b**) the interlayer Co thickness *t*_Co_ for the samples of [Co0.28/Ni0.58]_5_/Co(*t*_Co_)/Tb_30_Co_70_ (12).

Furthermore, we selected 12 nm-thick Tb_30_Co_70_ as the switching layer and investigated the PMA effect of the FM pinning layer on the *H*_ex_ and *H*_c⊥_. The perpendicular anisotropy energy *K*_p_ of the FM pinning layer was firstly modulated by changing the repetition number N of the [Co/Ni]_N_ multilayer. Figure 5(a) shows the *H*_c⊥_ and *H*_ex_ of the heterostructure, as well as the effective uniaxial anisotropy energy *K*_p_ of the single Co/Ni layer as a function of N for the [Co/Ni]_N_/Tb_30_Co_70_ (12.0) samples. The *K*_p_ was calculated according to *K*_p_ = *M*_*s*_*H*_*k*_/2, where *H*_*k*_ is the saturation magnetic field of in-plane magnetic loops. Apparently, the *K*_p_ increases monotonically with N due to the increased Co/Ni interfaces. From the maximum *H*_c⊥_ we can conclude that the magnetization is compensated at N = 4. Therefore, the magnetization of Co/Ni is dominant and the TbCo layer will be switched at N > 4. Although the magnetization and layer thickness of TbCo layer are fixed, we can still see an obvious *H*_ex_ increase, which can be ascribed to the enhanced *K*_p_ of the FM layer. By optimizing the layer structure, we achieved large *H*_c⊥_ up to 1.31 T and *H*_ex_ of 2.19 T simultaneously at the N = 4 case, which will be of great importance for practical applications. In addition, the PMA strength of Co/Ni layer was manipulated by tuning the thickness of an additional Co interlayer inserted between the [Co/Ni]_5_ and Tb_30_Co_70_ (12.0) layers as well. The interlayer Co thickness *t*_Co_ is kept below 1.2 nm to ensure the easy axis of Co/Ni along perpendicular direction. A maximum *K*_p_ occurs at *t*_Co_ = 0.28 nm, further increasing *t*_Co_ will give rise to *K*_p_ decrease. Such non-monotonic variation is the result of competition between the interfacial PMA and in-plane shape anisotropy. For this sample structure, the net magnetization is always dominated by [Co/Ni]/Co and increases with *t*_Co_, thus leading to the monotonic reduction of *H*_c⊥_, as shown in Fig. 5(b). Meanwhile, the *H*_ex_ follows a similar variation trend to the *K*_p_, again demonstrating that the exchange coupling field is strongly dependent on *K*_p_ of the pinning layer.

In conclusion, we have investigated the antiferromagnetic exchange coupling interactions and net magnetization switching in perpendicular [Co/Ni]_N_/TbCo composite structures. The magnetization compensation composition, compensation thickness of FM or FI layer, as well as compensation temperature for the coupled heterostructures have been clarified. By controlling the magnetization, thickness and PMA strength of the FM and FI layers, a wide range of variation in both net magnetization switching field *H*_c⊥_ and exchange coupling field *H*_ex_ are realized. The calculated interfacial coupling strength at RT is as strong as 4.4 ± 0.3 mJ/m^2^, leading to a large *H*_ex_ value even exceeding 3.0 T, which is significantly higher than the normal FM/AFM systems. These results offer us valuable information for practical applications in data storage technology and profound understanding of the fundamental exchange coupling mechanism.

## Methods

Different series of samples, in a structure of glass/Ta(3)/Cu(3)/[Co0.28/Ni0.58]_N_/Co (*t*_Co_)/Tb_x_Co_100–x_ (12)/Ta(5) (layer thickness in unit of nm), were deposited sequentially at ambient temperature in a Kurt J. Lesker magnetron sputter system with a base pressure better than 1 × 10^−8^ Torr. The TbCo alloy layer was fabricated by co-sputtering from pure Tb and Co targets, their relative atomic concentration was controlled by varying the sputtering power of Tb and determined by X-ray Photoelectron Spectroscopy (XPS). Magnetic properties were characterized by Vibrating Sample Magnetometer and Physical Property Measurement System.

## Additional Information

**How to cite this article**: Tang, M. H. *et al.* Interfacial exchange coupling and magnetization reversal in perpendicular [Co/Ni]_N_ /TbCo composite structures. *Sci. Rep.* **5**, 10863; doi: 10.1038/srep10863 (2015).
